# The Impact of the COVID-19 Pandemic on Tobacco Treatment Program Implementation at National Cancer Institute-Designated Cancer Centers

**DOI:** 10.1093/ntr/ntac160

**Published:** 2022-07-02

**Authors:** Sarah D Hohl, Kimberly A Shoenbill, Kathryn L Taylor, Mara Minion, Gleneara E Bates-Pappas, Rashelle B Hayes, Margaret B Nolan, Vani N Simmons, Michael B Steinberg, Elyse R Park, Kimlin Ashing, Diane Beneventi, Lisa Sanderson Cox, Adam O Goldstein, Andrea King, Chris Kotsen, Cary A Presant, Scott E Sherman, Christine E Sheffer, Graham W Warren, Robert T Adsit, Jennifer E Bird, Heather D’Angelo, Michael C Fiore, Claire Van Thanh Nguyen, Danielle Pauk, Betsy Rolland, Nancy A Rigotti

**Affiliations:** Carbone Cancer Center, University of Wisconsin-Madison, Madison, WI, USA; Department of Family Medicine, Lineberger Comprehensive Cancer Center, University of North Carolina-Chapel Hill, Chapel Hill, NC, USA; Georgetown University Medical Center, Lombardi Comprehensive Cancer Center, Cancer Prevention and Control Program, Washington, DC, USA; Carbone Cancer Center, University of Wisconsin-Madison, Madison, WI, USA; Department of Psychiatry and Behavioral Sciences, Memorial Sloan Kettering Cancer Center, New York, NY, USA; Department of Psychiatry, Massey Cancer Center, Virginia Commonwealth University, Richmond, VA, USA; Department of Population Health Sciences, University of Wisconsin School of Medicine and Public Health, Madison, WI, USA; Center for Tobacco Research and Intervention, University of Wisconsin School of Medicine and Public Health, Madison, WI, USA; Department of Health Outcomes and Behavior, Moffitt Cancer Center, Tampa, FL, USA; Center for Tobacco Studies, Cancer Institute of New Jersey, Rutgers Robert Wood Johnson Medical School, New Brunswick, NJ, USA; Department of Psychiatry, Massachusetts General Hospital (MGH), Boston, MA, USA; Department of Population Sciences, Center of Community Alliance for Research & Education, City of Hope National Medical Center, Duarte, CA, USA; Tobacco Research and Treatment Program, MD Anderson Cancer Center, University of Texas, Houston, TX, USA; Cancer Prevention and Control, University of Kansas School of Medicine, University of Kansas Cancer Center, Kansas City, KS, USA; Department of Family Medicine, Lineberger Comprehensive Cancer Center, University of North Carolina-Chapel Hill, Chapel Hill, NC, USA; Department of Psychiatry and Behavioral Neuroscience, University of Chicago, Chicago, IL, USA; Department of Psychiatry and Behavioral Sciences, Memorial Sloan Kettering Cancer Center, New York, NY, USA; Department of Medical Oncology and Therapeutics Research, City of Hope National Medical Center, Duarte, CA, USA; Department of Population Health, NYU Grossman School of Medicine, New York, NY, USA; Department of Health Behavior, Roswell Park Comprehensive Cancer Center, Buffalo, NY, USA; Department of Radiation Oncology, Medical University of South Carolina, Hollings Cancer Center, Charleston, SC, USA; Department of Cell and Molecular Pharmacology and Experimental Therapeutics, Medical University of South Carolina, Hollings Cancer Center, Charleston, SC, USA; Center for Tobacco Research and Intervention, University of Wisconsin School of Medicine and Public Health, Madison, WI, USA; Carbone Cancer Center, University of Wisconsin-Madison, Madison, WI, USA; Carbone Cancer Center, University of Wisconsin-Madison, Madison, WI, USA; Carbone Cancer Center, University of Wisconsin-Madison, Madison, WI, USA; Center for Tobacco Research and Intervention, University of Wisconsin School of Medicine and Public Health, Madison, WI, USA; Carbone Cancer Center, University of Wisconsin-Madison, Madison, WI, USA; Carbone Cancer Center, University of Wisconsin-Madison, Madison, WI, USA; Carbone Cancer Center, University of Wisconsin-Madison, Madison, WI, USA; Institute for Clinical and Translational Research, University of Wisconsin-Madison, Madison, WI, USA; Department of Medicine, Division of General Internal Medicine and Mongan Institute, Tobacco Research and Treatment Center, Harvard Medical School, Massachusetts General Hospital, Boston, MA, USA

## Abstract

**Introduction:**

The COVID-19 pandemic disrupted cancer screening and treatment delivery, but COVID-19’s impact on tobacco cessation treatment for cancer patients who smoke has not been widely explored.

**Aims and Methods:**

We conducted a sequential cross-sectional analysis of data collected from 34 National Cancer Institute (NCI)-designated cancer centers participating in NCI’s Cancer Center Cessation Initiative (C3I), across three reporting periods: one prior to COVID-19 (January–June 2019) and two during the pandemic (January–June 2020, January–June 2021). Using McNemar’s Test of Homogeneity, we assessed changes in services offered and implementation activities over time.

**Results:**

The proportion of centers offering remote treatment services increased each year for Quitline referrals (56%, 68%, and 91%; *p* = .000), telephone counseling (59%, 79%, and 94%; *p* = .002), and referrals to Smokefree TXT (27%, 47%, and 56%; *p* = .006). Centers offering video-based counseling increased from 2020 to 2021 (18% to 59%; *p* = .006), Fewer than 10% of centers reported laying off tobacco treatment staff. Compared to early 2020, in 2021 C3I centers reported improvements in their ability to maintain staff and clinician morale, refer to external treatment services, train providers to deliver tobacco treatment, and modify clinical workflows.

**Conclusions:**

The COVID-19 pandemic necessitated a rapid transition to new telehealth program delivery of tobacco treatment for patients with cancer. C3I cancer centers adjusted rapidly to challenges presented by the pandemic, with improvements reported in staff morale and ability to train providers, refer patients to tobacco treatment, and modify clinical workflows. These factors enabled C3I centers to sustain evidence-based tobacco treatment implementation during and beyond the COVID-19 pandemic.

**Implications:**

This work describes how NCI-designated cancer centers participating in the Cancer Center Cessation Initiative (C3I) adapted to challenges to sustain evidence-based tobacco use treatment programs during the COVID-19 pandemic. This work offers a model for resilience and rapid transition to remote tobacco treatment services delivery and proposes a policy and research agenda for telehealth services as an approach to sustaining evidence-based tobacco treatment programs.

## Introduction

Smoking is the leading cause of cancer and cancer death in the United States.^1^ Over 60% of cancer patients are current or former commercial tobacco smokers, and only 12% of patients who smoke quit within two years following a cancer diagnosis.^2^ Tobacco dependence treatment services offered as part of routine cancer care contributes to improved cancer and other health outcomes.^3^ However, until only a decade ago, only 60% of National Cancer Institute (NCI)-designated cancer centers reported offering any type of tobacco treatment to patients who smoke.^4^

To address this gap in cancer care, in 2017, the NCI established the Moonshot-funded Cancer Center Cessation Initiative (C3I). Three successive cohorts totaling 52 NCI-designated cancer centers and a coordinating center were funded to integrate and enhance evidence-based tobacco treatment into routine oncology care.^5^ Cohort 1 was funded from 2017 to 2019; Cohort 2 was funded from 2018 to 2020, and Cohort 3 was funded from 2020 to 2021. Participating C3I centers refer patients and deliver evidence-based counseling and pharmacotherapy using multiple modalities (eg, Quitline, in-person counseling). These tobacco treatment programs (TTPs) have increased the proportion of patients who receive tobacco treatment at NCI-designated cancer centers.^6^

The COVID-19 pandemic disrupted cancer care workflows for screening and treatment delivery,^7^ and for tobacco treatment within those contexts.^8^ Healthcare systems were forced to rapidly transition from providing in-person care to offering remote services via telephone or video,^9,10^ practices bolstered by the Centers for Medicaid and Medicare Services (CMS) new regulations that allow billing for remote services.^9^ Remote tobacco treatment services offered during COVID-19 have been found to be feasible and acceptable and result in high patient engagement.^8^ Broader investigation of the impact of COVID-19 on tobacco treatment delivery in cancer care and the role of telehealth in supporting sustained TTP implementation is needed to guide the investment of resources to support new and existing telehealth programs with the goal of improving cancer and tobacco-related health outcomes among patients who smoke. In this sequential cross-sectional analysis, we examined TTP service delivery—including telehealth practices—and implementation activities among C3I cancer centers before and during the COVID-19 pandemic.

## Methods

Every six months, C3I centers report the progress of their TTP to the C3I coordinating center. For this analysis, we summarized data from 34 of the 52 centers that completed reports for three six-month periods, each one year apart: one prior to the pandemic (January–June 2019) and two during the pandemic—the first as the virus emerged in the United States (January–June 2020) and the second later in the pandemic (January–June 2021). Centers that did not report data across all three reporting periods were excluded (*n* = 18, including 10 centers that were newly funded beginning in late 2020). First, we sought to identify changes in tobacco treatment services implemented and referrals provided between 2019 and 2021, including telephone- and video-based counseling practices (Supplementary Table S1). Second, we aimed to assess TTP implementation activities most and least impacted by COVID-19 (Table 1).

**Table 1.** Proportion of C3I Centers Reporting High Impact of COVID-19 on Implementation Activities in Two Reporting Periods During the Pandemic (*n* = 34 Centers)

	Highly impacted	Highly impacted	*p*
**Implementation activity**	Jan–June 2020 (%)	Jan–Jun 2021 (%)	
Expand to additional clinics/sites	62	53	.549
Enroll tobacco users into treatment	59	50	.453
Modify the clinical workflow	56	38	.625
Maintain staff and clinician morale and enthusiasm	53	12	**.001**
Hire new program staff	50	44	.625
Screen and identify tobacco users for treatment	47	38	.375
Train clinicians/staff	44	29	.125
Provide/deliver cessation counseling	35	29	.687
Conduct follow up with patients	32	27	.687
Modify the electronic health record	29	24	.625
Provide pharmacotherapy	27	24	1.000
Generate electronic health record reports for program evaluation	27	24	1.000
Refer to external services (e.g., Quitline, Smokefree TXT)	24	15	.250
Maintain current program staff	15	15	1.000

These data were only collected during the COVID-19 pandemic, and consequently are not available for reporting periods prior to February 2020.

To meet the second analytic aim, starting in 2020, additional items were added to the routine report to assess provision of video-based counseling and the degree to which key implementation activities were affected by COVID-19. For the latter, we added 14 Likert items with five response options ranging from “not at all” to “a great amount”. For analytic and interpretive simplicity, we created dichotomous variables for analysis: “minimally impacted” (“little/no impact” responses) and “highly impacted” (“moderate/a lot/a great amount” responses).

Data were uploaded into SPSS version 27 for analysis. We summarized descriptive statistics to describe changes over time prior to and during COVID-19 in TTP activities, staffing, and services and referral, including telehealth use. We conducted a within-subjects z-test for equality of proportions, using McNemar’s Test of Homogeneity to determine if there was a change in the types of treatment offered from 2019 to 2021. We applied the same analysis to assess changes in the impact of COVID-19 on implementation activities between 2020 and 2021. The University of Wisconsin-Madison IRB deemed this study routine program evaluation and IRB-exempt.

## Results

### Tobacco Treatment Services, Including Telephone and Video-based Telehealth

**Figure 1.**
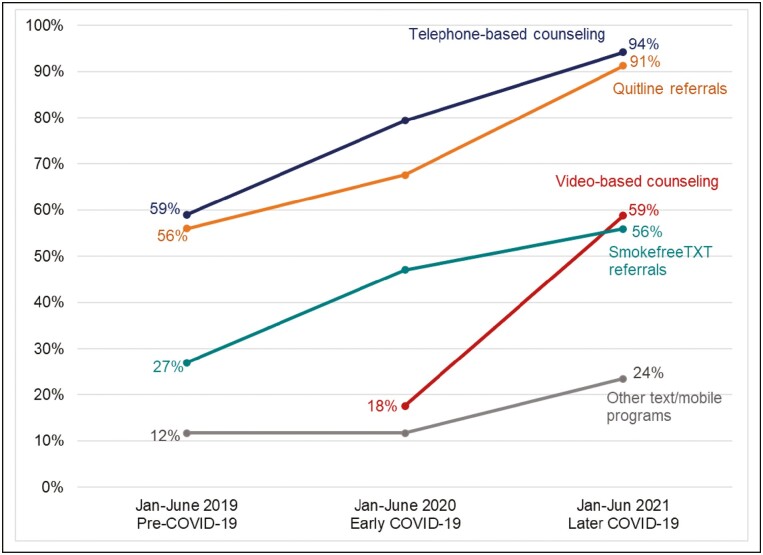
Change over time in tobacco cessation services and modalities offered between Jan–June 2019 and Jan–June 2021 at NCI-designated cancer centers (*n* = 34). Figure 1 illustrates the five services with the greatest change over time. See Supplemental Table 1 for change over time for all services and referrals offered.

Figure 1 illustrates the five tobacco treatment services with the greatest change across participating sites between January–June 2019 and January–June 2021. Supplementary Table S1 reports changes across all three reporting periods for all services offered across C3I centers. Between 2019 and 2021, the proportion of C3I centers that offered referrals to a Quitline, a telephone-based resource not associated with the patient’s clinic or health system, increased from 56% to 91% (*p* < .001). Referrals to Smokefree TXT, a free mobile text message service that supports those who want to quit smoking, increased from 27% to 56% (*p* = .006). Provision of clinic- or health-system–based group and individual telephone counseling increased from 59% to 94% (*p* = .002). Video-based counseling was not reported in 2019; 6 centers (18%) offered that service in 2020 and 20 (59%) did so in 2021 (*p* = .006), with 14 of these (70%) reporting having done so in response to COVID-19. Nine (28%) of the 32 centers that offered telephone-based counseling in 2021 reported establishing that modality in response to COVID-19. Across centers and reporting periods, the most widely and consistently offered treatments were cessation medication and individual counseling (delivered via any modality).

Of the 32 centers that offered telephone-based counseling in 2021, only 7 centers billed for that service; 26 centers reported they plan to continue to offer the service independent of reimbursement, while 6 centers indicated the continuation of the service will be contingent on reimbursement. Of the 20 sites that offered video-based counseling, 10 billed for the service, and 12 centers said they would continue to offer the service independent of reimbursement by insurers.

### TTP Implementation Activities

#### Staff Retention and Training

The number of centers that reported laying off or furloughing staff decreased from 3 (8.8%) in 2020 to 1 (2.9%) in 2021, and the mean level of full-time equivalents (FTE) for tobacco treatment specialists decreased slightly from 1.9 to 1.7.

#### Activities Reported by Centers as Being Most Impacted Across COVID-19 Reporting Periods

Table 1 reports the proportion of centers that reported a high impact on implementation activities during the COVID-19 pandemic. In 2020, the majority of centers reported the following activities were highly affected by COVID-19: expanding to additional clinics, enrolling new tobacco users into treatment, modifying the clinical workflow, maintaining staff and clinical morale, and hiring new program staff. In contrast, by 2021 only two activities were highly affected by COVID-19 at the majority of C3I centers: expanding to additional clinics and enrolling new tobacco users into treatment.

#### Activities Reported by Centers as Being Minimally Impacted Across COVID-19 Reporting Periods

In 2020, the majority of centers reported the following activities as minimally affected by COVID-19: modifying the EHR, providing pharmacotherapy, generating EHR reports for program evaluation, referring to external services, and maintaining current program staff. In 2021, the same activities continued to be minimally impacted by COVID-19 across all centers, with the addition of maintaining staff and clinician morale.

#### Activities Reported by Centers as Having the Greatest Change From 2020 to 2021

Compared to 2020, in 2021, more centers reported minimal impacts of COVID-19 on implementation activities. In both years, only 15% of centers indicated that COVID-19 had a high impact on maintaining program staff (*p* = 1.00). In 2020, 53% of centers reported that maintaining staff and clinician morale was highly impacted by COVID-19, but that proportion decreased to 12% by 2021 (*p* = .001). Between 2020 and 2021, centers reporting high impact on training clinicians and staff decreased from 44% to 29% (*p* = .125), and those reporting high impact on modifying the clinical workflow decreased from 56% to 38% (*p* = .625).

## Discussion

We assessed changes in TTP delivery across three years, starting before COVID-19 emerged (2019) to one year after (2021), and examined the impact of COVID-19 on tobacco treatment implementation activities across 34 NCI-designated cancer centers, including telehealth practices as a key component of sustaining TTPs during the pandemic. Centers reported few adverse effects overall on delivering evidence-based tobacco treatment to patients who smoke, likely made possible by centers’ abilities to maintain staffing levels, consistently provide training, and increase telephone- and video-based care and Quitline referrals. A year after the virus emerged, centers displayed evidence of their resilience. They had adapted to implementation challenges, reporting fewer morale issues, and workflow difficulties, and a greater ability to train staff and screen and refer tobacco users to treatment than the previous year.

Pre-pandemic, 59% of C3I centers already had a telephone-based tobacco treatment counseling component. However, the emergence of COVID-19 necessitated rapid scale-up of these remote programs and the addition of video-based services across C3I centers, as COVID-19 severely reduced the availability of in-person healthcare visits. Although the effectiveness of telephone counseling for smoking cessation has been established,^11^ studies comparing whether video telehealth offers an advantage over telephone care have been conflicting.^12^ The COVID-19 pandemic presents opportunities to assess whether and to what extent video telehealth for smoking cessation is effective and if it adds benefit beyond telephone care.

Although CMS payment regulations have enabled billing for remote services during the pandemic^9^ a minority of C3I centers bill for remote tobacco treatment services, despite cancer centers’ widespread adoption of telehealth within and beyond their TTPs.^13^ This could be due to difficulties with reimbursement for services depending on which provider offers treatment, or insurance policies regarding the number and volume of tobacco treatment services covered. Policies regarding continued reimbursement for remote services and impact on patient care will influence if and how telehealth is sustained over time. Future research could investigate best practices for billing for tobacco cessation in cancer care.

Evidence demonstrates effectiveness of remote cessation services among patients with cancer.^14^ However, increased disparities in telehealth use have been documented among racial and ethnic minoritized patients and those living in rural areas,^15,16^ who may have low digital literacy and limited access to a stable broadband connection and electronic devices (ie, phone, tablet, computer). Provider-level barriers to telehealth have also been reported, including differing opinions among medical oncology providers as to the benefits and barriers to telemedicine visits and unwillingness to engage with patients on these platforms.^17^ Incorporating training for patients on what to expect during telemedicine visits and for behavioral health and oncology providers to deliver telemedicine effectively will maximize the quality and effectiveness of this visit mode.^18^ These known barriers must be addressed to ensure telehealth services reach patients equitably and mitigate rather than exacerbate health disparities. Moreover, innovative approaches are needed to evaluate the ability of telehealth services to improve access, efficacy, and equity of tobacco cessation treatment among cancer patients,^13^ including access and utilization among patients from minoritized backgrounds.

## Limitations

There are several limitations to this work. First, the increase in tobacco treatment services offered by programs between 2019 and 2021 cannot solely be attributed to the COVID-19 pandemic, as program development in the C3I initiative was simultaneously ongoing. Second, the 34 sites that reported at all three timepoints may represent those best positioned to respond to COVID-19. Ten of the 18 centers not included in the analysis were part of Cohort 3, whose first reporting period was January–June 2021. The remaining 8 centers were all located in large metropolitan areas, including one in the South, 3 in the Midwest, and 2 on the east coast. C3I does not collect patient-level data or catchment area characteristics, so it is difficult to determine whether these centers served a greater number of underserved patients. However, the diversity of the 34 centers, which vary in geographic location, size, patient population, and length of time implementing TTPs, represents a strength of this work. Third, the impact of the rapid shift to providing increased telehealth counseling and Quitline referrals on program reach and effectiveness was not assessed; however, this work is underway as part of C3I evaluation activities. Fourth, although we documented the number of programs that offered specific services, we do not have information on whether the volume of services changed. Although NCI, Commission on Cancer, and the American Association for Cancer Research recommend including screening for and treating tobacco use as a quality measure,^19^ reporting for tobacco use screening and cessation is not required of cancer centers. As a large implementation initiative, the burden of data collection must be weighed against centers’ capacity to simultaneously collect and report data and provide services. Thus, as an initiative, C3I does not collect data on volume of services provided or patient-level data, including clinical and patient-reported outcomes. Future research is needed to compare tobacco treatment telehealth use by different cancer patient population subgroups and to assess patient-level smoking cessation outcomes by modality (ie, telephone vs. video). In addition, investigating how patient choice impacts telehealth program reach and effectiveness will be critical to designing effective and adaptive TTPs for patients with cancer.

## Conclusions

The COVID-19 pandemic necessitated rapid transition to new telehealth program delivery of tobacco treatment to patients with cancer. C3I cancer centers adjusted promptly to challenges, with improvements reported in staff morale and ability to train providers, refer patients to tobacco treatment, and modify clinical workflows. These factors enabled C3I centers to sustain evidence-based tobacco treatment implementation during and beyond the COVID-19 pandemic. The observation that reduced expansion to additional clinics was one of the components most adversely impacted by the pandemic emphasizes that to reach more smokers, and to do so equitably, program and institutional leaders should consider planning expansion efforts. Since the concordance of smoking, cancer, and COVID-19 has been described as a “tragic triad” with markedly adverse outcomes for cancer patients,^20^ continuing and improved support for tobacco control programs should be imperative for institutions and health care systems.

## Supplementary Material

A Contributorship Form detailing each author’s specific involvement with this content, as well as any supplementary data, are available online at https://academic.oup.com/ntr.

ntac160_suppl_Supplementary_Table_S1

ntac160_suppl_Supplementary_Taxonomy-form

## Data Availability

The data underlying this article cannot be shared publicly as permission was not granted from the cancer centers participating in the Cancer Center Cessation Initiative. The data may be shared on reasonable request to the Cancer Center Cessation Initiative Coordinating Center.
