# Supplementary material for: The Impact of the COVID-19 Pandemic on Tobacco Treatment Program Implementation at National Cancer Institute-Designated Cancer Centers
Source: Nicotine Tob Res. 2022 Jul 2;25(2):345–9. doi: 10.1093/ntr/ntac160 (PMC9384385; doi:10.1093/ntr/ntac160)
Supplement: ntac160_suppl_Supplementary_Table_S1 [file ntac160_suppl_supplementary_table_s1.docx]

**Supplemental Table 1**. Tobacco treatment services offered, Jan-June 2019 to Jan-June 2021 (n=34 centers)

| Tobacco Treatment Service | Jan-June 2019 | Jan-June 2020 | Jan-Jun 2021 | | p- value |
| --- | --- | --- | --- | --- | --- |
| Video-based counseling* |  | 18% | 59% | | **0.006** |
| Quitline (fax or eReferral) | 56% | 68% | 91% | | **0.000** |
| Telephone-based counseling | 59% | 79% | 94% | | **0.002** |
| SmokefreeTXT (with or without eReferral) | 27% | 47% | 56% | | **0.006** |
| Individual counseling | 68% | 82% | 94% | | 0.219 |
| Other text/mobile program | 12% | 12% | 24% | | 0.344 |
| Cessation Medication | 85% | 88% | 88% | | 1.000 |
| TelASK/IVR | 12% | 15% | 15% | | 0.500 |
| Group counseling | 32% | 32% | 29% | | 0.727 |
| Web resource (e.g., Smokefree.gov) | 38% | 35% | 29% | | 0.581 |
| Video-based and telephone-based treatment include group or individual, non-automated, and not Quitline counseling | | | | | |
| *Video-based counseling was not assessed in 2019 | | | |  |  |
| p-value is based on McNemar's Test of Homogeneity comparing 2019 and 2021; changes in video-based counseling implementation compared between 2020 and 2021 | | | | | |
